# Evolutionary Plasticity of AmrZ Regulation in *Pseudomonas*

**DOI:** 10.1128/mSphere.00132-18

**Published:** 2018-04-18

**Authors:** David A. Baltrus, Kevin Dougherty, Beatriz Diaz, Rachel Murillo

**Affiliations:** a School of Plant Sciences, University of Arizona, Tucson, Arizona, USA; University of Iowa

**Keywords:** AmrZ, motility, Pseudomonas stutzeri

## Abstract

*amrZ* encodes a master regulator protein conserved across pseudomonads, which can be either a positive or negative regulator of swimming motility depending on the species examined. To better understand plasticity in the regulatory function of AmrZ, we characterized the mode of regulation for this protein for two different motility-related phenotypes in Pseudomonas stutzeri. As in Pseudomonas syringae, AmrZ functions as a positive regulator of swimming motility within P. stutzeri, which suggests that the functions of this protein with regard to swimming motility have switched at least twice across pseudomonads. Shifts in mode of regulation cannot be explained by changes in AmrZ sequence alone. We further show that AmrZ acts as a positive regulator of colony spreading within this strain and that this regulation is at least partially independent of swimming motility. Closer investigation of mechanistic shifts in dual-function regulators like AmrZ could provide unique insights into how transcriptional pathways are rewired between closely related species.

**IMPORTANCE** Microbes often display finely tuned patterns of gene regulation across different environments, with major regulatory changes controlled by a small group of “master” regulators within each cell. AmrZ is a master regulator of gene expression across pseudomonads and can be either a positive or negative regulator for a variety of pathways depending on the strain and genomic context. Here, we demonstrate that the phenotypic outcomes of regulation of swimming motility by AmrZ have switched at least twice independently in pseudomonads, so that AmrZ promotes increased swimming motility in P. stutzeri and P. syringae but represses this phenotype in Pseudomonas fluorescens and Pseudomonas aeruginosa. Since examples of switches in regulatory mode are relatively rare, further investigation into the mechanisms underlying shifts in regulator function for AmrZ could provide unique insights into the evolution of bacterial regulatory proteins.

## Abstract (precis)

Microbes often display finely tuned patterns of gene regulation across different environments, with major regulatory changes controlled by a small group of “master” regulators within each cell. AmrZ is a master regulator of gene expression across pseudomonads and can be either a positive or negative regulator for a variety of pathways depending on the strain and genomic context. Here, we demonstrate that the phenotypic outcomes of regulation of swimming motility by AmrZ have switched at least twice independently in pseudomonads, so that AmrZ promotes increased swimming motility in P. stutzeri and P. syringae but represses this phenotype in Pseudomonas fluorescens and Pseudomonas aeruginosa. Since examples of switches in regulatory mode are relatively rare, further investigation into the mechanisms underlying shifts in regulator function for AmrZ could provide unique insights into the evolution of bacterial regulatory proteins.

## INTRODUCTION

Transcriptional regulation of bacterial operons is often tightly balanced to enable rapid phenotypic changes while minimizing energetic costs associated with overproduction of mRNA and proteins (1–3). Indeed, survival of bacterial populations requires gene regulatory schemes that can respond to fine-scale gradients and rapid shifts in environmental conditions, and data suggest that these regulatory schemes are optimized to appropriately respond to a variety of possible environments (1, 4, 5). Dissection of the mechanisms of action and characterization of transcriptional responses for numerous repressors and activators across species has provided knowledge about the functions of these proteins while also creating a context for exploring how such pathways evolve (4–6). Although there exists great appreciation for the general plasticity of bacterial regulatory networks, our understanding of how particular pathways are transcriptionally rewired and how specific proteins change regulatory mode over evolutionary time remains far from complete. While much previous work has focused on defining how and when genes are regulated, there have been few examples that have pinpointed changes in the direction of regulation by the same protein across closely related lineages. Here, we further document an example in pseudomonads involving the ribbon-helix-helix transcriptional regulator AmrZ.

*amrZ*, alginate and motility regulator Z, encodes a ribbon-helix-helix transcription factor that directly regulates a variety of pathways across *Pseudomonas* species and is a master regulator for numerous pathways associated with virulence in P. aeruginosa (7). Unlike the vast majority of characterized regulators, AmrZ can directly affect transcription both positively and negatively within the same cell, with the precise function depending on the intrinsic structure of the protein and the genomic context of the DNA binding site (8, 9). Moreover, although the mechanism of repression by AmrZ canonically involves binding to operator regions, how this protein specifically activates transcription remains unclear (8, 9). Regulatory pathways involving AmrZ are best described for Pseudomonas aeruginosa, where expression from *amrZ* is promoted by AlgT (10), activates 9 genes, and represses 49 genes, including itself (7). Regulation of AmrZ can also be affected posttranscriptionally by a small RNA, *ersA* (11). Pathways directly repressed by AmrZ include swimming motility and the operon controlling production of the exopolysaccharide Psl (7). Interestingly, AmrZ can differentially modulate construction of all three type VI secretion systems in P. aeruginosa (12). In contrast, AmrZ has been shown to promote expression of the alginate operon and type IV pilus-dependent twitching motility (13, 14). AmrZ has also been shown to influence colony morphology and biofilm formation, with these phenotypes mediated by AmrZ-dependent changes in cyclic di-GMP (7). In Pseudomonas fluorescens F113, *amrZ* mutants are hypermotile in swimming assays and iron uptake genes are derepressed (15), with many of these phenotypes appearing to be modulated by changes in cyclic di-GMP (16). In Pseudomonas syringae DC3000, AmrZ is a positive regulator of swimming motility as well as a variety of other virulence genes (17).

Pseudomonas stutzeri is an environmentally ubiquitous species known best as a denitrifier as well as for its diverse metabolic capabilities (18). Phylogenies of pseudomonads demonstrate that P. stutzeri is placed uniquely in between other species where impacts of AmrZ on swimming motility have been evaluated (see reference 19 and Fig. 4), and thus, information about the role of AmrZ from this species could polarize our understanding of regulatory modes for this protein. Given opposing information about the regulatory role of AmrZ across pseudomonads, the original aim of this paper was to test how AmrZ regulated swimming motility in P. stutzeri. We subsequently noticed that AmrZ also positively regulates a colony spreading phenotype in this species and that an allele of this gene enables the differentiation of positive regulation for these two phenotypes. Our results therefore provide a new viewpoint for understanding how regulatory pathways can evolve in bacteria.

## RESULTS AND DISCUSSION

**FIG 1:**
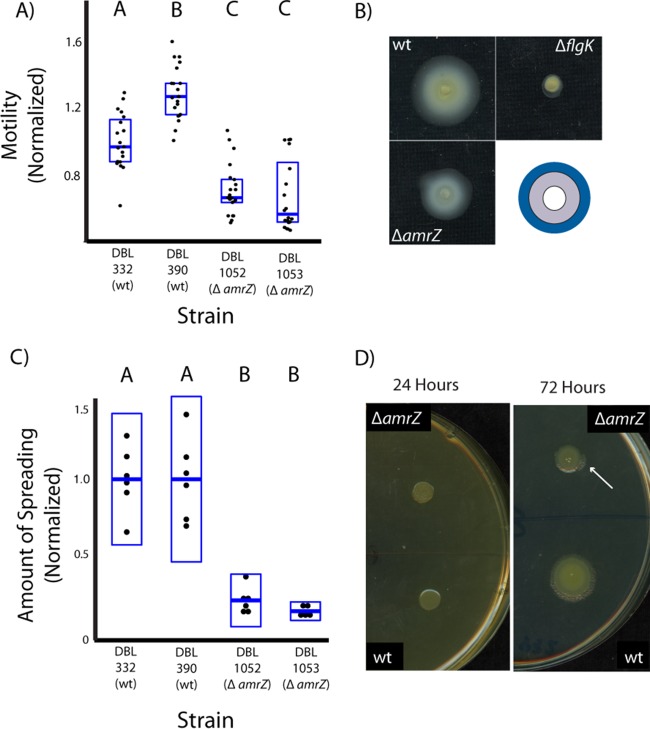
*amrZ* is a positive regulator of swimming motility and colony spreading in Pseudomonas stutzeri. Strains DBL1052 and DBL1053 are derived from strains DBL332 and DBL390, respectively, and contain independently created deletions in *amrZ*. Individual data points for each assay are plotted for each strain, with boxes representing 2 standard deviations and means plotted as horizontal blue lines at the center of the box plots. Measurements within each assay have been normalized so that the value of DBL332 is 1. Letters above each box plot indicate that mean values are significantly different at *P* < 0.01 according to Tukey’s HSD test. (A) AmrZ is a positive regulator of swimming motility, and loss of swimming motility is seen in independently created *amrZ* deletion lines. (B) Representative images from motility assays are shown for a wild-type strain, an *amrZ* deletion strain, and an *flgK* deletion strain. In each case, colored circles have been created that match halos in these pictures and have been superimposed in the final image (blue, DBL332; gray, DBL1052; white; DBL1168). (C) AmrZ is a positive regulator of colony spreading, and loss of spreading is seen in independently created *amrZ* deletion lines. (D) Representative example of colony spreading activity and positive regulation by AmrZ. The same plate is shown after being scanned after 1 day of growth (left) and after 3 days of growth (right). AmrZ^+^ strains spread outward on KB medium over time, while AmrZ^−^ strains fail to spread unless there are compensatory mutations (as shown by the white arrow in the DBL1052 strain). wt, wild type.

**FIG 2:**
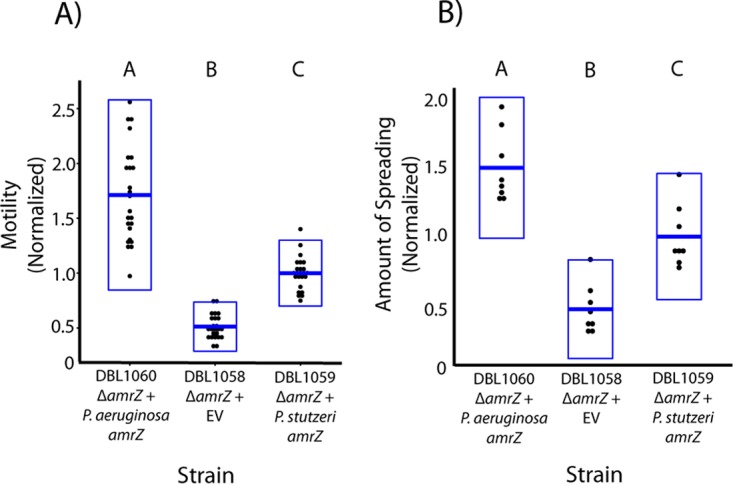
Alleles of *amrZ* from either P. stutzeri or P. aeruginosa can phenotypically complement an *amrZ* deletion strain in *trans*. Strains DBL1058, DBL1059, and DBL1060 are derived from strain DBL830 (Δ*amrZ*). DBL1058 contains an empty-vector gentamicin resistance cassette, while DBL1059 contains *amrZ* (native promoter) integrated into the Tn*7* site of the chromosome. DBL1060 contains the *amrZ* allele from P. aeruginosa (native promoter from P. stutzeri) integrated into the Tn*7* site of the chromosome. Individual data points for each assay are plotted for each strain, with boxes representing 2 standard deviations and means plotted as horizontal blue lines at the center of the box plots. Motility and colony spreading values are normalized so that the value of DBL1059 is 1. Letters above each box plot indicate that mean values are significantly different at *P* < 0.01 according to Tukey’s HSD test. Swimming motility (A) and colony spreading (B) phenotypes in an *amrZ* deletion strain can be complemented by alleles of *amrZ* from either P. stutzeri or P. aeruginosa.

AmrZ is a well-studied regulator of phenotypes important for environmental survival across pseudomonads, including multiple virulence traits in P. aeruginosa (7, 15, 17). Since P. stutzeri is uniquely phylogenetically positioned among the strains previously characterized for AmrZ-dependent regulation of motility, we investigated phenotypic effects of deleting *amrZ* in this bacterium. In both deletion lines (DBL1052 and DBL1053), loss of *amrZ* leads to decreased flagellar motility, clearly demonstrating that AmrZ is a positive regulator for this phenotype within this strain (Fig. 1A). However, that these lines display relatively higher motility than an immotile *flgK* deletion strain demonstrates that AmrZ is not the sole positive regulator for flagellar operons in P. stutzeri (Fig. 1B). Loss of motility can be complemented by expression of *amrZ* with its native promoter from the Tn*7* site (Fig. 2A), and we note that complemented strains have levels of swimming motility at least equal to that of the wild type (https://figshare.com/s/2a79a1e48fa851d51577). This result directly contrasts with what has been reported across multiple other *Pseudomonas* species (10, 15) but supports the role of AmrZ as a positive regulator of motility in P. syringae (17). Using these same strain comparisons, we have also been able to show that AmrZ is a positive regulator of colony spreading in P. stutzeri (Fig. 1C and D and 2B).

At this time, it is difficult to discern which molecular changes underlie shifts in AmrZ function in P. stutzeri because the mechanism of regulation by this protein for motility differs across pseudomonads. In P. aeruginosa, AmrZ binds upstream of *fleQ* in order to directly repress expression of *fleQ*; however, no AmrZ binding was observed upstream of *fleQ* in P. fluorescens F113 even though *amrZ* mutants are hypermotile (7, 15). As a first step to determine the molecular mechanism behind alterations in AmrZ-dependent regulation of motility, we tested whether the P. aeruginosa allele could complement the loss of motility in P. stutzeri. These alleles are slightly diverged from one another but overall maintain relatively high sequence similarity (see Fig. 4C), and our assays thus test whether a small number of protein structural changes are directly responsible for regulatory shifts in phenotypes. As shown in Fig. 2A and B, the P. aeruginosa allele of *amrZ* is able to complement both the swimming motility and colony spreading defects in P. stutzeri, demonstrating that the two versions of this protein regulate motility in a similar way. Therefore, it does not appear that the phenotypic switch in regulation by AmrZ is due to amino acid changes in the protein itself, which strongly suggests that differential regulation of motility across these strains is mediated by loci downstream of AmrZ regulation. It is also possible that changes within AmrZ-interacting proteins could explain differences in phenotype, but there are currently no proteins known to interact in this way. We also note that, although the initial results of this experiment suggest that the P. aeruginosa allele could be hyperactive, we have created independent versions of each of these strains and find that there is no consistent difference in motility between the two alleles of *amrZ* during complementation at the Tn*7* site (https://figshare.com/s/2a79a1e48fa851d51577).

**FIG 3:**
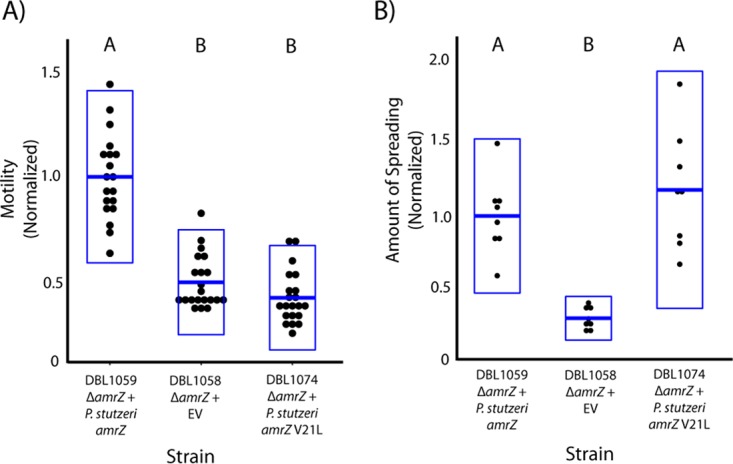
AmrZ independently regulates swimming motility and colony spreading. Strains DBL1058, DBL1059, and DBL1074 are derived from strain DBL830 (Δ*amrZ*). DBL1058 contains an empty-vector gentamicin resistance cassette, while DBL1059 contains *amrZ* (native promoter) integrated into the Tn*7* site of the chromosome. DBL1074 contains a mutant version of *amrZ* (AmrZV21L) integrated into the Tn*7* site of the chromosome. Individual data points for each assay are plotted for each strain, with boxes representing 2 standard deviations and means plotted as horizontal blue lines at the center of the box plots. Motility and colony spreading values are normalized so that the value of DBL1059 is 1. Letters above each box plot indicate that mean values are significantly different at *P* < 0.01 according to Tukey’s HSD. (A) The wild-type version of AmrZ can complement the swimming motility defect of DBL830, but the AmrZV21L mutant version cannot. (B) Both the wild-type version of AmrZ and the AmrZV21L mutant version can complement a colony spreading deficiency of DBL830.

**FIG 4:**
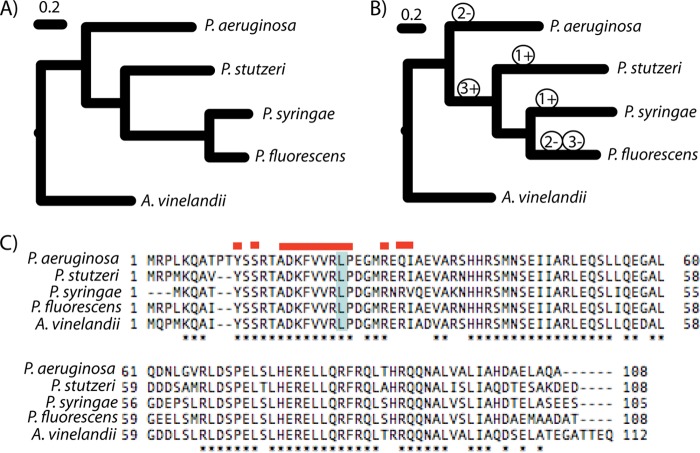
The mode of regulation of AmrZ in swimming motility has shifted at least twice across *Pseudomonas*. (A and B) Bayesian phylogenies were built using either RpoD/GyrB (A) or AmrZ (B) for four strains of *Pseudomonas* for which the role of AmrZ in swimming motility has been evaluated and using Azotobacter vinelandii as an outgroup. Support for all nodes on each phylogeny is >0.95 posterior probability, and the phylogeny of RpoD/GyrB matches that built from whole-genome information (data not shown). (B) Three equally parsimonious scenarios, labeled 1 to 3, for the evolution of mode of regulation of AmrZ for swimming motility are overlaid onto the phylogeny of AmrZ. “+” indicates that positive regulation arose during each of the three scenarios, while “−” indicates that negative regulation arose. Parsimonious inferences follow logically from an assumption that AmrZ is a negative regulator of swimming motility (scenarios 1 and 3) or a positive regulator (scenario 2) in A. vinelandii. Given these starting assumptions, the number of regulatory switches required to explain the phenotypic data given phylogenies was deduced by hand. (C) Protein alignments of AmrZ for the strains used in phylogenetic comparisons are shown. Red lines above this alignment indicate amino acids that have been shown to be involved in dimerization of AmrZ (according to reference 8). We also highlight, in light blue, the amino acid that has changed in the AmrZV21L allele.

Interestingly, we observed that a mutant allele of *amrZ* (AmrZV21L) arose and swept to fixation during an ongoing evolutionary passage experiment using P. stutzeri and carried out within our lab (data not shown; see also Fig. 4C). This mutation occurs within a region of AmrZ known to be involved in dimerization in P. aeruginosa (8) and therefore possibly affects interactions between independent copies of this protein in P. stutzeri. Surprisingly, we demonstrate here that the mutant AmrZV21L allele is able to complement the colony spreading phenotype but not the swimming motility phenotype within our *amrZ*-knockout strains (Fig. 3). This result suggests that, even though colony spreading and swimming motility are both positively regulated by AmrZ within P. stutzeri, the mechanisms of positive regulation for these two phenotypes are at least partially independent. We do not yet know how regulation of these pathways mechanistically differs, but the position of the mutation suggests that dimerization is required only for positive regulation of swimming motility. Recent demonstrations have shown that AmrZ can indirectly alter pools of cyclic di-GMP (7, 16), and since cyclic di-GMP is a critical signaling molecule for some types of motility across pseudomonads (20), it is also possible that regulatory independence of these phenotypes reflects differential influence of cyclic di-GMP. At the very least, that these two phenotypes are independently regulated by AmrZ speaks to the evolutionary flexibility of positive regulation by AmrZ.

Given the challenges inherent in defining precise types of bacterial motility, it is possible that the colony spreading observed in this report is phenotypically correlated with twitching or swarming motility. P. stutzeri* amrZ* deletion mutant colonies appear “rough” and “dry” during growth on King’s B (KB) medium compared to wild-type strains but are as competent for natural transformation on saltwater LB medium (suggesting a functional pilus [21]) as wild-type strains (data not shown). It is therefore likely that phenotypic differences in colony spreading in the *amrZ* mutants are due to lack of production of molecules like surfactants rather than direct changes in either flagellar or pilus-dependent motility.

To demonstrate possible evolutionary scenarios explaining differential regulation of swimming motility by AmrZ across pseudomonads, we inferred phylogenies by using critical strains for which swimming motility effects of AmrZ have been evaluated as well as an outgroup (Azotobacter vinelandii) that also contains a version of this regulator. As one can see in Fig. 4B, there are three equally parsimonious scenarios for the evolution of positive regulation of swimming motility by AmrZ. Under the first scenario (labeled 1), AmrZ is a negative regulator of swimming motility in the ancestor of pseudomonads, and positive regulation has independently evolved twice. Under the second scenario (labeled 2), the ancestral version of AmrZ is a positive regulator of swimming motility, and negative regulation has independently evolved twice. Under the third scenario, AmrZ is a negative regulator of swimming motility in an ancestral strain, evolves to be a positive regulator before the split of P. stutzeri and P. syringae, and subsequently evolves as a negative regulator again in P. fluorescens. That the phylogeny of AmrZ matches that of RpoD/GyrB, coupled with the ability of the P. aeruginosa allele to complement P. stutzeri phenotypes, rules out scenarios implicating horizontal gene transfer of *amrZ* in changes to the mode of regulation for swimming motility. In any case, this example definitively demonstrates that the mode of regulation for AmrZ for swimming motility has changed at least twice independently.

Only a few bacterial transcriptional regulators are known to act as both activators and repressors of gene expression. One of these proteins, AmrZ, has been canonically considered a negative regulator of motility across pseudomonads with one exception shown to date (7, 15, 17). We provide evidence for the evolutionary plasticity of AmrZ, by demonstrating that this protein acts independently as a positive regulator of swimming motility and colony spreading phenotypes in P. stutzeri. Therefore, we definitively show that there have been at least two independent shifts in function for AmrZ in the context of swimming motility across pseudomonads. Closer investigation of mechanistic shifts in dual-function regulators like AmrZ could provide unique insights into how transcriptional pathways are rewired between closely related species.

## MATERIALS AND METHODS

### Bacterial strains, plasmids, and culture conditions.

All strains and plasmids used in the study are listed in Table 1. DBL332 was selected as a rifampin-resistant isolate of strain 23a24 (22). Strain propagation of P. stutzeri largely took place at 27°C in King’s B medium supplemented with rifampin. Antibiotics were used in the following concentrations where appropriate: 50 µg/ml rifampin, 10 µg/ml tetracycline, 40 µg/ml nitrofurantoin, 10 µg/ml gentamicin, and 20 µg/ml kanamycin.

**TABLE 1:** Strains and plasmids

Strain or plasmid number	Strain or plasmid description	Citation
Strains		
DBL332	P. stutzeri DBL332, Rif^r^	22
DBL390	P. stutzeri DBL390, Rif^r^ Gent^r^ LacZ^+^	22
DBL830	P. stutzeri DBL332 with pDBL64 integrated, Rif^r^ Tet^r^ Suc^s^	This work
DBL831	P. stutzeri DBL390 with pDBL64 integrated, Rif^r^ Gent^r^ LacZ^+^ Tet^r^ Suc^s^	This work
DBL1052	P. stutzeri DBL332 with deletion of *amrZ*, Rif^r^	This work
DBL1053	P. stutzeri DBL390 with deletion of *amrZ*, Rif^r^ Gent^r^ LacZ^+^	This work
DBL1058, DBL1259	P. stutzeri DBL1052 with Tn*7* transposition from pME3280a, Rif^r^ Gent^r^	This work
DBL1059, DBL1262	P. stutzeri DBL1052 with Tn*7* transposition from pDBL93, Rif^r^ Gent^r^	This work
DBL1060, DBL1252	P. stutzeri DBL1052 with Tn*7* transposition from pDBL94, Rif^r^ Gent^r^	This work
DBL1074	P. stutzeri DBL1052 with Tn*7* transposition from pDBL95, AmrZL21V, Rif^r^ Gent^r^	This work
DBL1118	P. stutzeri DBL332 with pDBL99 integrated, Rif^r^ Tet^r^ Suc^s^	This work
DBL1168	P. stutzeri DBL332 with deletion of *flgK*, Rif^r^	This work
Plasmids		
pME3280a	Tn*7* empty vector	28
Tn*7*-GW	Promoterless Gateway destination vector for Tn*7* transposition	J. Chang, unpublished data
pDBL63	P. stutzeri* amrZ* deletion construct in pDONR207	This work
pDBL64	P. stutzeri* amrZ* deletion construct in pMTN1907	This work
pDBL91	P. stutzeri* amrZ* ORF with stop codon and P. stutzeri promoter in pDONR207	This work
pDBL92	P. aeruginosa* amrZ* ORF with stop codon and P. stutzeri promoter in pDONR207	This work
pDBL93	P. stutzeri* amrZ* ORF with stop codon and P. stutzeri promoter in Tn*7*-GW	This work
pDBL94	P. aeruginosa* amrZ* ORF with stop codon and P. stutzeri promoter in Tn*7*-GW	This work
pDBL95	P. stutzeri* amrZC61G* ORF with stop codon and P. stutzeri promoter in pDONR207	This work
pDBL96	P. stutzeri* amrZC61G* ORF with stop codon and P. stutzeri promoter in Tn*7*-GW	This work
pDBL99	P. stutzeri* flgK* deletion construct in pDONR207	This work
pDBL100	P. stutzeri* flgK* deletion construct in pMTN1907	This work
pMTN1907	Gateway destination vector for generating deletions in P. stutzeri	23

### Creation of *amrZ* mutants.

Creation of the construct for deleting *amrZ* is described in depth at https://doi.org/10.6084/m9.figshare.3204178.v1. Briefly, regions upstream and downstream of *amrZ* in P. stutzeri DBL332 were amplified and spliced using overlap PCR. This fragment was recombined into pDONR207 using BP recombinase to create pDBL63. pDBL63 was then recombined with pMTN1907 to create pDBL64, which can be used to cleanly delete gene regions within P. stutzeri (22, 23). Once pDBL64 was created, it was mated into either DBL332 or DBL390 through triparental mating with the helper strain containing plasmid pRK2013 (DAB42). DBL390 is a gentamicin-resistant version of DBL332 where *lacZ* has been integrated into the Tn*7* site using pUC18-mini-Tn*7*T-Gm-LacZ prior to conjugation with pDBL64 and therefore represents independent deletion of *amrZ*. After mating, tetracycline-resistant recombinants in both DBL332 and DBL390 backgrounds were selected on LB medium. Each recombinant was grown overnight in LB medium supplemented with rifampin and spread on KB plates containing 5% sucrose. Isolates of P. stutzeri DBL332 and DBL390 in which *amrZ* has been deleted are distinguishable when grown on KB medium because they manifest as rough colonies. Clean deletion of *amrZ* was confirmed through PCR with primers DBL383 and DBL384.

### Complementation of *amrZ*.

Creation of complementation constructs is described at https://doi.org/10.6084/m9.figshare.3365263.v1. Briefly, the *amrZ* open reading frame (ORF) (including the stop codon) and native promoter for *amrZ* were amplified from strain DBL332. This fragment was purified and recombined into pDONR207 using BP Clonase to create pDBL91. This construct was then recombined from pDBL91 into a Tn*7* transposon on the Gateway destination vector pTn*7*-GW (J. Chang, unpublished data) to create pDBL93. Last, this construct was transposed onto the DBL1052 chromosome (a clean deletion of *amrZ* in the DBL332 background) through natural transformation after mixing cells with both pDBL93 and pTNS2. The complementation construct for the mutant allele of *amrZ* (DBL1074, AmrZV21L) was created using these same primer sets by amplifying this region from a strain naturally containing the allele and using BP Clonase to create plasmid pDBL95. The resulting destination vector from this construct (pDBL96) was used for transposition in the same way as the wild-type version described above. A complementation construct using the P. aeruginosa *amrZ* ORF was created in a similar way except that the BP reaction was carried out with a synthesized gBlock (Integrated DNA Technologies, Inc., Coralville, IA) to create pDBL92. Expression of the P. aeruginosa allele of *amrZ* in this construct is driven by the same promoter sequence (from P. stutzeri) as in pDBL93. pDBL94 was created through an LR reaction involving pDBL92 and pTn*7*-GW and transformed into DBL1052.

### Creation of *flgK* mutant strain.

In order to test whether a knockout of *amrZ* was partially motile or completely immotile, we constructed a strain of P. stutzeri (DBL1157) in which *flgK* had been completely deleted from the genome. A file describing the creation of the *flgK* mutant can be found at https://figshare.com/s/a9b633c3dcb7acbadcfe.

### Motility assays.

Following overnight growth in LB medium and two washes with 1 ml 10 mM MgCl_2_, the optical density at 600 nm (OD_600_) of each strain was standardized at 1.0 in 10 mM MgCl_2_. A blunt-ended toothpick was dipped into this inoculum and then dipped into the center of a 12-well tissue culture plate containing one-half-strength LB medium with 0.25% agar. Plates were covered with Parafilm and incubated for either 24 or 48 h at room temperature (indicated in figure legends), after which they were scanned at 600 dots per inch (dpi). Images were imported into ImageJ, and the pixel area of each strain was quantified. Data for all assays can be found at https://figshare.com/s/bd8d3d0a12b8c9f36b5b. The motility of each strain for each assay was normalized to a control strain within the same experiment, with figure legends identifying the normalized strain, so that motilities across experiments were comparable. Normalization did not affect overall statistical outcomes (data not shown). Each set of experiments was independently run twice, with at least 8 replicates per experiment. Statistical tests were carried out in R (24), with one-way analyses of variance (ANOVAs) with “normalized motility” as the dependent variable and “strain” as a fixed-effect independent variable. Inclusion of “assay” as a second independent variable did not affect overall statistical outcomes (data not shown). Tukey’s honestly significant difference (HSD) test was then used to classify strain effects.

### Colony spreading assays.

Following overnight growth in KB medium and two washes with 1 ml 10 mM MgCl_2_, the OD_600_ of each strain was standardized at 1.0 in 10 mM MgCl_2_. Ten microliters of this suspension was pipetted onto King’s B (KB) medium with 1.5% agar. Each experimental plate contained all strains within a given comparison (Fig. 1C). Plates were covered in Parafilm and incubated for 72 h at room temperature. At three time points (after 24, 48, and 72 h of growth), plates were scanned at 600 dpi. Images were imported into ImageJ, and the pixel area of each strain was quantified. Data for all assays can be found at https://figshare.com/s/bd8d3d0a12b8c9f36b5b. The amount of spreading was calculated by taking the difference in area between 24 and 72 hours for each strain on each plate, except for one case where spreading was calculated between 48 and 72 hours. Spreading of each strain for each assay was normalized to a strain from the same plate within the same experiment, with figure legends identifying the normalized strain, so that motilities across experiments were comparable. Normalization did not affect overall statistical outcomes (data not shown). Each set of experiments was independently run twice, with at least 2 (but usually 4) replicated plates per experiment. Statistical tests were carried out in R (24), with one-way ANOVAs with “normalized spreading” as the dependent variable and “strain” as a fixed-effect independent variable. Inclusion of “assay” as a second independent variable did not affect overall statistical outcomes (data not shown). Tukey’s HSD test was then used to classify strain effects.

### Phylogenetic comparisons.

Bayesian phylogenies were created using protein sequences from conserved genes from each strain, obtained from the JGI Integrated Microbial Genomes (IMG) database (25). Strains used for this comparison were P. stutzeri 23a24 (IMG identifier [ID] 2565956579), P. fluorescens F113 (IMG ID 2511231156), P. syringae pv. tomato DC3000 (IMG ID 2508501074), P. aeruginosa PAO1 (IMG ID 637000218), and Azotobacter vinelandii CA (IMG ID 2541047084).

Protein sequences for GyrB and RpoD were used to infer phylogeny of “housekeeping” genes. GyrB and RpoD sequences were independently aligned using Clustal X (26) and then concatenated. MrBayes was used for Bayesian phylogenetic analysis on these sequences (27), using flat priors and a burn-in period of 25,000 generations. In each case, convergence of the run occurred before 100,000 total generations. Phylogenies built using whole-genome sequences for these strains completely agree with the reported trees (data not shown). A phylogeny for AmrZ was built in the same way as that of RpoD/GyrB. Alignments and output files from MrBayes can be found on Figshare at https://figshare.com/s/bd8d3d0a12b8c9f36b5b.

### Data availability.

Data underlying all statistical analyses, sequence alignments, and evolutionary analyses within this paper are available through Figshare at https://doi.org/10.6084/m9.figshare.3365299.v4 and https://doi.org/10.6084/m9.figshare.5965417.v1.
